# Supplementary material for: Dissociating the behavioral and computational features of implicit motor learning and explicit perturbation detection
Source: bioRxiv. 2026 Jun 28:2026.06.25.734533. Preprint. [Version 1] doi: 10.64898/2026.06.25.734533 (PMC13320948; doi:10.64898/2026.06.25.734533)
Supplement: 1 [file NIHPP2026.06.25.734533v1-supplement-1.pdf]

bioRxiv preprint doi: https://doi.org/10.64898/2026.06.25.734533; this version posted June 28, 2026. The copyright holder for this preprint (which was not certified by peer review) is the author/funder, who has granted bioRxiv a license to display the preprint in perpetuity. It is made available under a CC-BY 4.0 International license.

Supplement

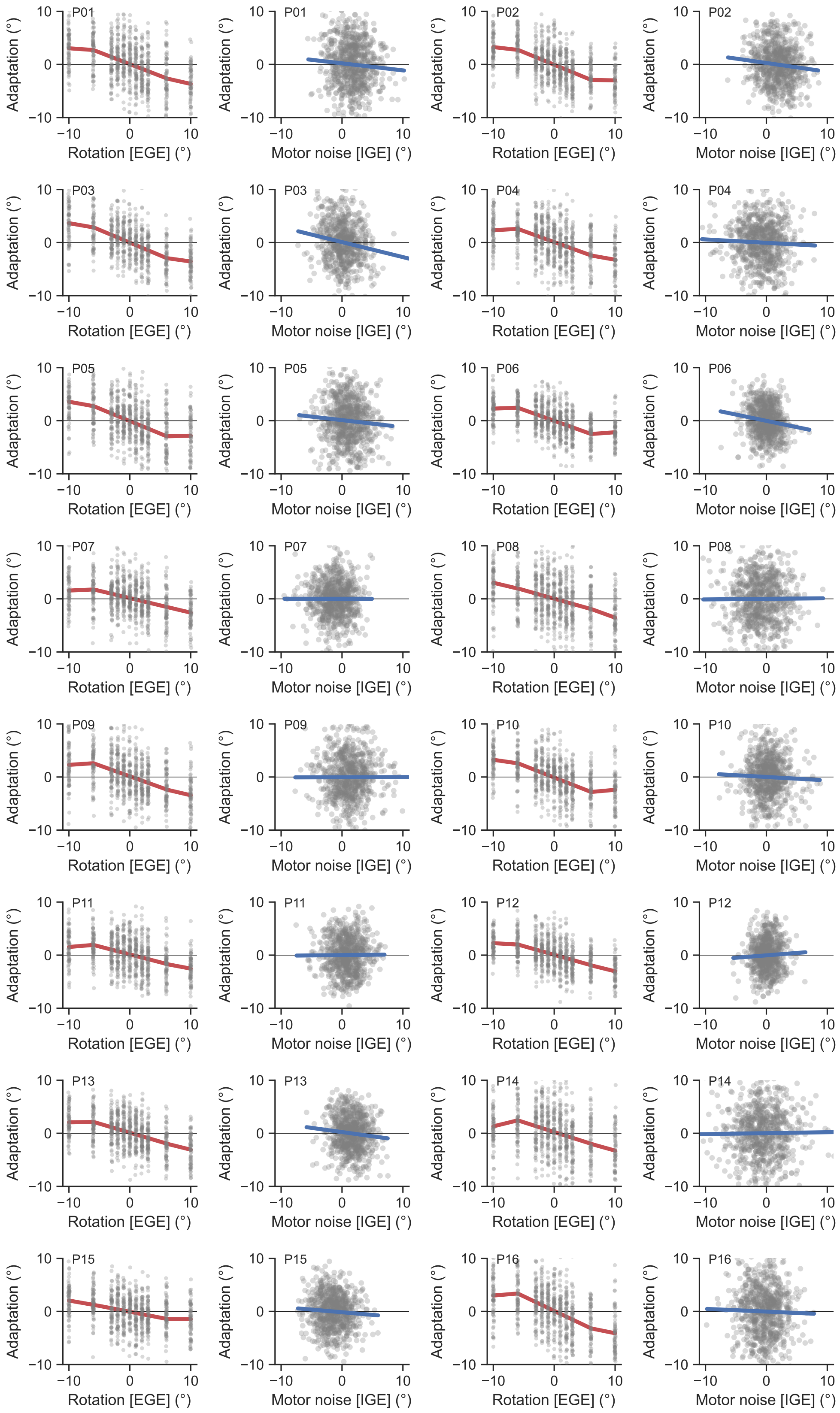

Figure 7: Plots of individual data (as in Fig. 2) of all 16 participants from the adaptation task.

bioRxiv preprint doi: https://doi.org/10.64898/2026.06.25.734533; this version posted June 28, 2026. The copyright holder for this preprint (which was not certified by peer review) is the author/funder, who has granted bioRxiv a license to display the preprint in perpetuity. It is made available under a CC-BY 4.0 International license.

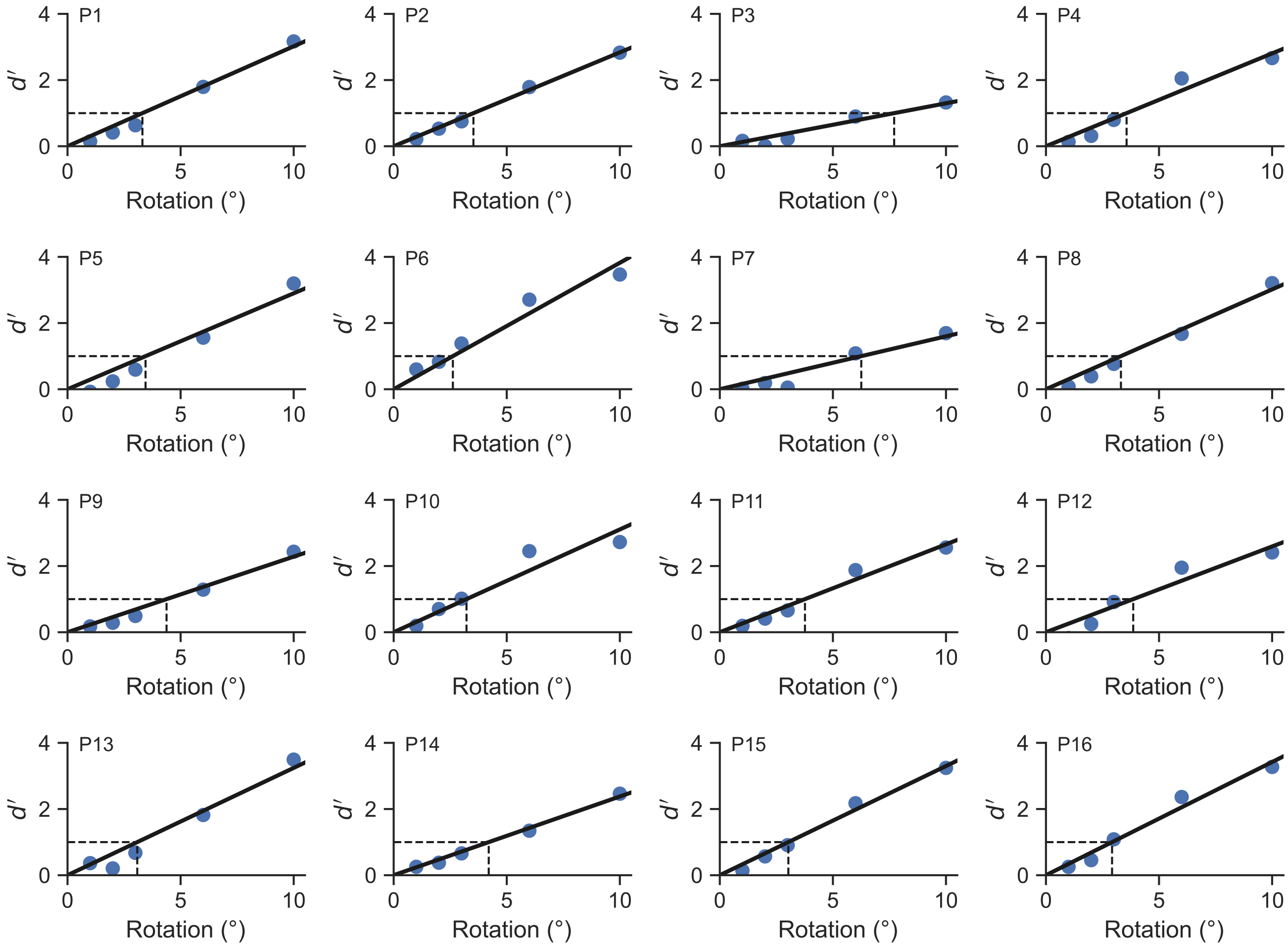

Figure 8: (Plots of individual data (as in Fig. 3) of all 16 participants from the signal-detection task.

bioRxiv preprint doi: https://doi.org/10.64898/2026.06.25.734533; this version posted June 28, 2026. The copyright holder for this preprint (which was not certified by peer review) is the author/funder, who has granted bioRxiv a license to display the preprint in perpetuity. It is made available under a CC-BY 4.0 International license.

806 ## Parameter Estimates

Model	Parameter	Mean [95% CI]
PIECE	α	0.571 [0.466, 0.676]
	β	0.246 [0.225, 0.268]
	σ_p	0.195 [0.125, 0.266]
	σ_{pert}	100.731 [85.621, 115.842]
	b	0.167 [-0.360, 0.695]
VCO	D	5.890 [4.283, 7.557]
	β	0.209 [0.188, 0.231]
	σ_v	6.630 [4.762, 8.499]
	b	0.170 [-0.335, 0.675]
CO	D	1.651 [0.897, 2.406]
	β	0.301 [0.271, 0.331]
	σ_{comb}	3.899 [2.575, 5.223]
	b	0.176 [-0.327, 0.679]

Table 1: Maximum-likelihood estimates of each model parameter during the adaptation task. Note: β in PIECE refers to the slope associated with increasing visual uncertainty whereas β in the VCO and CO models refer to the learning rate for adaptation.

Model	Parameter	Mean [95% CI]
PIECE	α	1.486 [1.074, 1.897]
	β	0.090 [0.225, 0.268]
	σ_p	3.289 [2.799, 3.779]
	σ_{pert}	4.540 [2.756, 6.324]
	b	-0.263 [-0.790, 0.254]
VCO	D	3.964 [3.589, 4.340]
	σ_v	2.469 [2.137, 2.801]
	b	-0.088 [-0.686, 0.509]
CO	D	3.475 [3.132, 3.817]
	σ_{comb}	2.881 [2.554, 3.222]

Table 2: Maximum-likelihood estimates of each model parameter during the signal-detection task.

bioRxiv preprint doi: https://doi.org/10.64898/2026.06.25.734533; this version posted June 28, 2026. The copyright holder for this preprint (which was not certified by peer review) is the author/funder, who has granted bioRxiv a license to display the preprint in perpetuity. It is made available under a CC-BY 4.0 International license.

807 **Model- and parameter-recovery analysis**

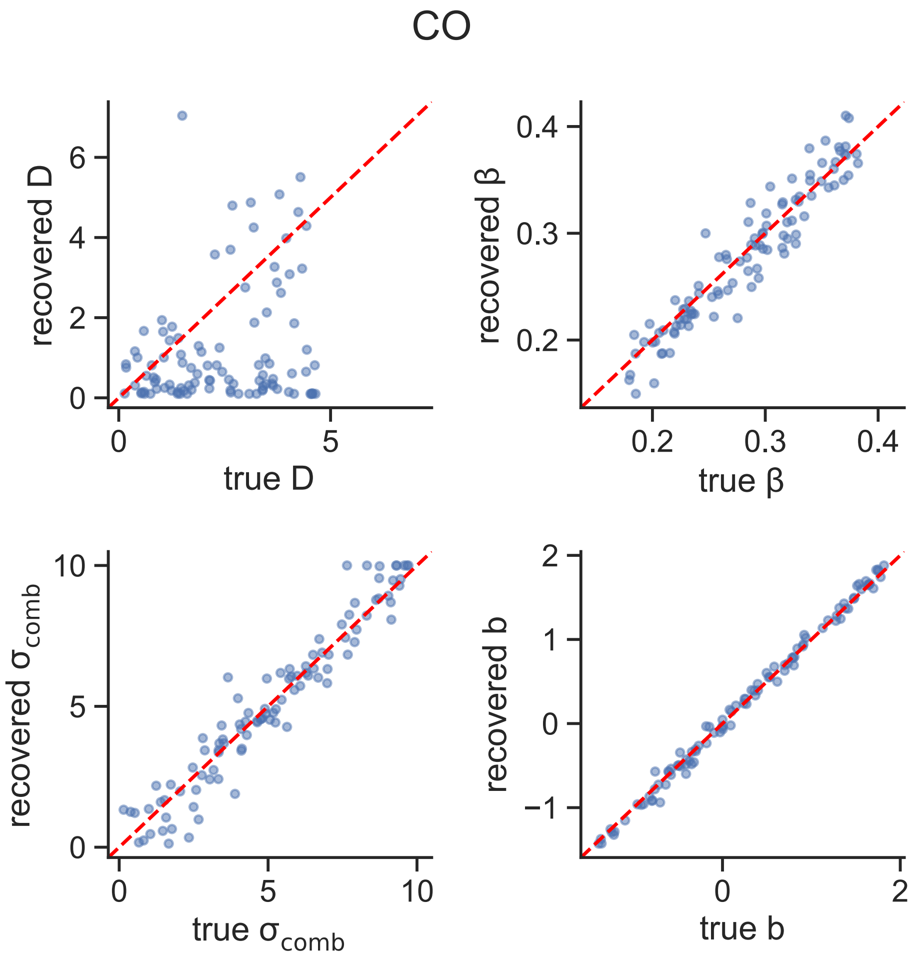

Figure 9: Parameter recovery for the winning CO model in the adaptation task. Scatterplots show the CO model parameter values that were recovered (y-axes) from fits to 100 synthetic datasets generated with the CO model. The parameter values used to generate the synthetic data are shown on the x-axes.

808 To validate the parameter values obtained by fitting CO or PIECE (i.e., the winning models for each task)
809 to our behavioral data and our ability to distinguish between each of the three models, we performed
810 parameter- and model-recovery analyses (R. C. Wilson and Collins, 2019). We generated 100 synthetic
811 datasets with each of the three models used in this study, totaling 300 datasets in all. The synthetic data
812 from each model were generated using parameter values drawn from uniform distributions bounded by the
813 minimum and maximum parameter values obtained from fits to each individual participant's behavioral data
814 (i.e., the range of maximum-likelihood estimates of each model parameter). We then fit all 300 synthetic
815 datasets with each model using maximum-likelihood estimation and performed objective model selection
816 for each dataset.

bioRxiv preprint doi: https://doi.org/10.64898/2026.06.25.734533; this version posted June 28, 2026. The copyright holder for this preprint (which was not certified by peer review) is the author/funder, who has granted bioRxiv a license to display the preprint in perpetuity. It is made available under a CC-BY 4.0 International license.

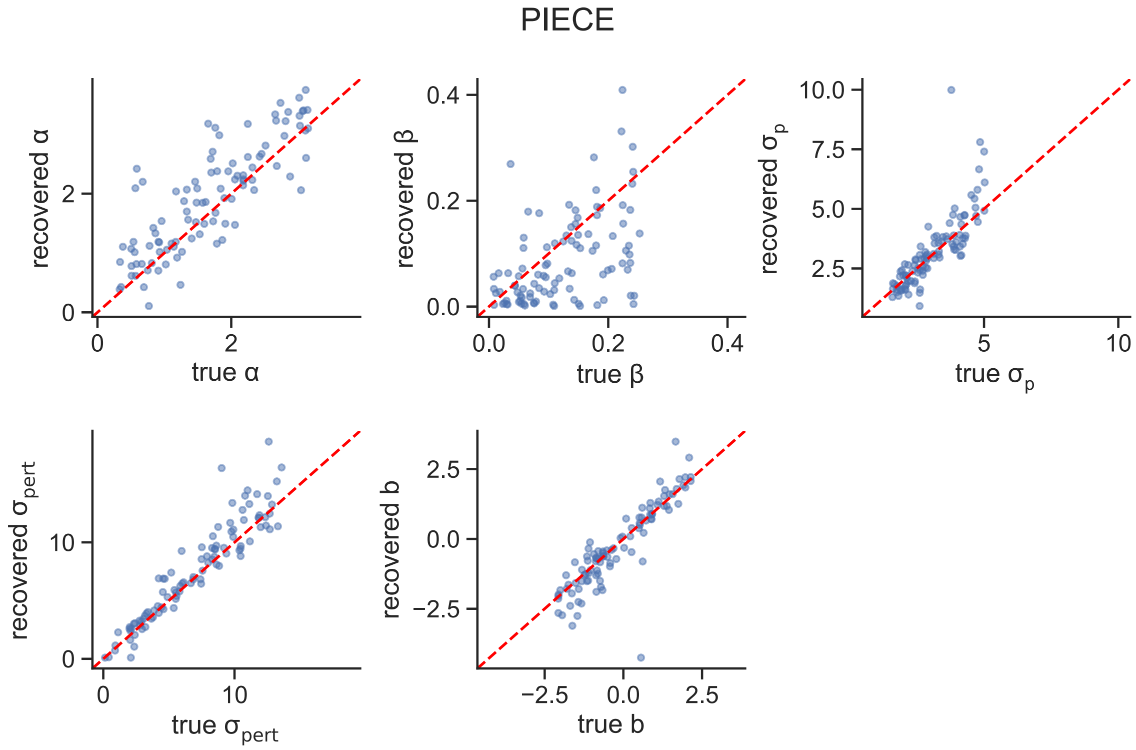

Figure 10: Parameter recovery for the winning PIECE model in the signal-detection task. Scatter plots show the PIECE model parameter values that were recovered (y-axes) from fits to 100 synthetic datasets generated with the PIECE model. The parameter values used to generate the synthetic data are shown on the x-axes.

bioRxiv preprint doi: https://doi.org/10.64898/2026.06.25.734533; this version posted June 28, 2026. The copyright holder for this preprint (which was not certified by peer review) is the author/funder, who has granted bioRxiv a license to display the preprint in perpetuity. It is made available under a CC-BY 4.0 International license.

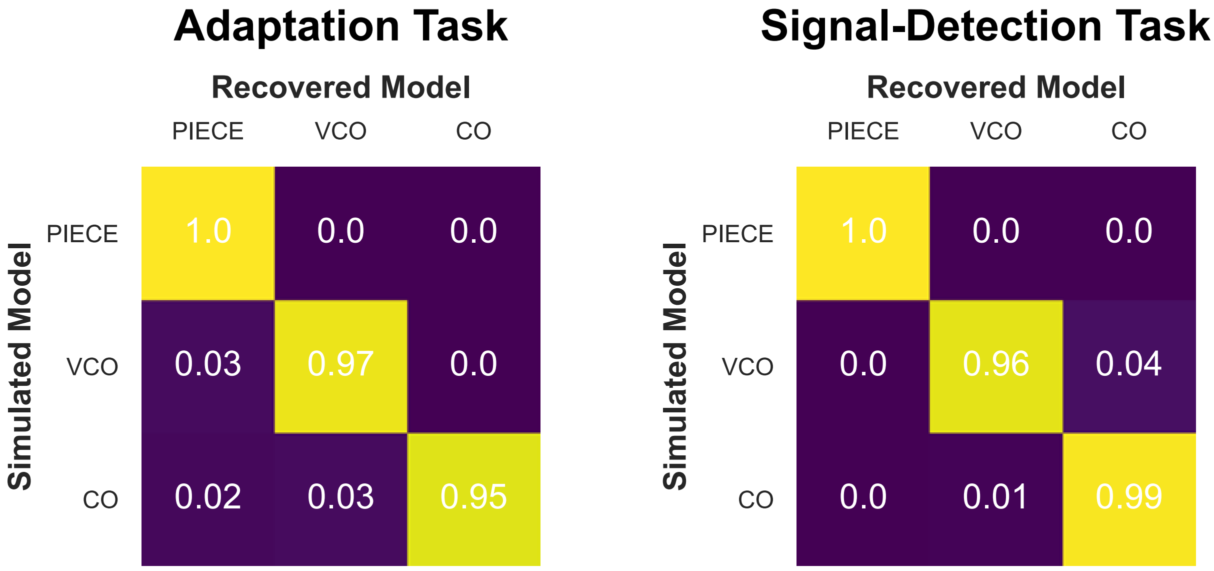

Figure 11: Confusion matrices for both tasks. The value in each cell of the matrix indicates p(recovered model | simulated model.

As shown in Figure 9, except for the detection threshold, D, the recovered parameters for the CO model were all highly correlated with the parameter values used to generate the synthetic data (Pearson's $r >= 0.953$, $p < 10^{-6}$). In the case of D, the correlation coefficient, r, was weaker ($r = 0.248$, $p = 0.013$). Future experiments that test a wider range of perturbation sizes will help achieve more accurate recovery of this parameter, as the current experiment spanned a relatively narrow range.

For the signal-detection task, the recovered parameters for the PIECE model were also, in general, tightly correlated with the parameter values used for simulation. The correlation coefficients, r, were all greater than 0.835, except in the case of β, the parameter representing the rate at which visual uncertainty increases as a function of visual cue distance from the target, which had a more moderate value of 0.493.

Most importantly, our model recovery analysis showed that we can accurately identify which of the three models was responsible for generating each synthetic data set with at least 95% accuracy. And in the case of the PIECE model, 100% accuracy across both tasks. This points to the robustness of our modeling results, as well as the strength of experimental design as we were successfully able to identify the model that generated the data in nearly all cases.
